# Growth Outcomes in Children with Familial Mediterranean Fever: A Question Beyond Chronic or Relapsing Inflammation

**DOI:** 10.3390/diseases14060186

**Published:** 2026-05-23

**Authors:** Ignazio Cammisa, Clelia Cipolla, Donato Rigante

**Affiliations:** 1 Department of Pediatrics, San Giovanni Evangelista Hospital, 00019 Tivoli, Italy; 2 Department of Life Sciences and Public Health, Fondazione Policlinico Universitario A. Gemelli IRCCS, 00168 Rome, Italy; clelia.cipolla@policlinicogemelli.it (C.C.); donato.rigante@unicatt.it (D.R.); 3 Periodic Fever Research Center, Università Cattolica Sacro Cuore, 00168 Rome, Italy

**Keywords:** Familial Mediterranean fever, growth, cytokines, interleukin-1, inflammation, children, colchicine, personalized medicine

## Abstract

*Background/Objective:* Familial Mediterranean fever (FMF) is an autoinflammatory disease caused by missense *MEFV* mutations, leading to recurrent episodes of interleukin (IL)-1β-mediated inflammation, and represents a model of cytokine-induced growth hormone (GH) resistance. Chronic or relapsing inflammatory bouts may impair growth in FMF children through functional alterations of the GH-insulin-like growth factor 1 (IGF-1) axis; however, the impact and reversibility of growth deficit remain unclear. The aim of this review is to assess data related to linear growth in young patients with FMF. *Methods:* This scoping review was conducted following PRISMA guidelines, searching for studies evaluating growth outcomes in FMF via the PubMed database. Fourteen studies, including 1144 children, were analyzed, evaluating height, growth velocity, IGF-1 levels, and treatment effects of colchicine or IL-1–targeted biologics. *Results:* Growth was generally preserved in a considerable number of children with FMF. Longitudinal analyses showed improvement in height standard deviation scores (HSDS) along with earlier and higher cumulative doses of colchicine. FMF attack frequency and overall disease severity modestly seemed to influence growth, whereas inflammatory markers were inconsistently correlated with growth parameters. Biologic therapies targeting IL-1 (canakinumab and anakinra) also showed positive effects on HSDS. Children with specific *MEFV* variants (such as M694V) or higher disease activity scores were at risk of developing a subtle growth impairment. *Conclusions:* Data on final height, though limited, suggest the preservation of growth in most pediatric patients with FMF. The maintenance of a normal linear growth is related to regular treatment with colchicine, though IL-1 blockers also appear to be beneficial in refractory FMF cases. These data highlight the importance of periodic, proactive check-ups and regular growth monitoring in children with FMF.

## 1. Introduction

Systemic autoinflammatory syndromes are either inherited or acquired disorders of innate immunity characterized by recurrence of seemingly unprovoked febrile attacks of different duration with multi-system inflammation of variable severity: the vast majority of these conditions, when observed in pediatrics is caused by mutations in genetic systems involved in the orchestration of inflammation and apoptosis, including hereditary inflammasomopathies, pyogenic disorders, immune-mediated granulomatous diseases, and interferon-signed syndromes [1]. Familial Mediterranean fever (FMF) is a prototypical autoinflammatory disease, and its Mendelian disease transmission occurs in an autosomal recessive mode; patients with FMF display self-limited episodes of fever and sterile inflammation, particularly affecting serosal membranes, joints, skin or other tissues, with 75–90% of cases presenting before age 20 [2]. Pediatric presentations are broader in comparison with those in adults, making it challenging to make a definite diagnosis of FMF. This disease is caused by missense mutations in the *MEFV* (from *M*editerranean *F*e*V*er) gene, which are mostly located on exon 10 of the short arm of chromosome 16: the gene encodes a 781-amino acid protein known as “pyrin”, also named “marenostrin,” referring to the Latin name for the Mediterranean Sea, once called ‘*mare nostrum*’. Pyrin is composed of five domains, each with a distinct role, and plays a decisive role in innate immunity as a structural component of the pyrin inflammasome, resulting in cleavage of the latent pore-forming protein gasdermin D and pro-inflammatory cytokines interleukin (IL)-1β and IL-18, but also regulating cytoskeleton signaling and specifically pyroptosis of blood cells [3]. In detail, mutations in the *MEFV* gene reduce the activation threshold of the pyrin inflammasome and lead to its inappropriate activation in the absence of infectious triggers, resulting in the recurrent inflammatory attacks that define the disease. IL-1β also triggers the release of further pro-inflammatory cytokines, including tumor necrosis factor (TNF)-α, IL-6, interferon (IFN)-γ, and IL-17, whose activity is balanced by IL-4 and IL-10, which display anti-inflammatory effects, influencing overall disease severity and expression.

Furthermore, dysregulation of IL-1 may persist between attacks, suggesting that subclinical inflammation fuels chronic consequences in patients with FMF in the long run [4]. More specifically, RhoA GTPase activates the serine-threonine kinases PKN1 and PKN2 that physiologically phosphorylate pyrin, which binds to the inhibitory chaperone 14-3-3 protein: this process maintains pyrin in an inactive state that prevents formation of the pyrin inflammasome. After pyrin dephosphorylation and detachment of the protein 14-3-3, there is a conformational change enabling pyrin interaction with the adapter protein ASC, which leads to pyrin inflammasome assembly and its final activation [5]. The other three autoinflammatory syndromes have been linked to inappropriate activation of the pyrin inflammasome: (a) pyrin-associated autoinflammation with neutrophilic dermatosis, (b) pyogenic arthritis, pyoderma gangrenosum and acne syndrome and (c) hyper-IgD syndrome, which is also known as mevalonate kinase deficiency [6,7,8]. Colchicine, a lipophilic alkaloid compound extracted by the lily *Colchicum autumnale*, is currently the mainstay of therapy for FMF: it disrupts microtubule polymerization and thereby affects pyrin inflammasome assembly and turns off cytokine secretion [9]. Classic FMF manifestations, characterized by recurrent episodes of fever and serositis (chest, abdomen, joints), leading to inflammatory attacks during childhood, may change according to the different national cohorts of studies, but both earlier disease onset and biallelic exon 10 *MEFV* mutations can influence disease severity, requiring colchicine dose optimization or IL-1 inhibitors [10].

Beyond the classical manifestations of FMF, chronic inflammation has important implications, particularly in children, as it can negatively affect growth and development. Indeed, recurring inflammatory flares, as observed in FMF, may impair growth plate development through both systemic and local effects on the growth hormone (GH) axis and cytokine output [11]. In particular, pro-inflammatory cytokines like IL-1 can interfere with multiple mechanisms, including GH and/or insulin-like growth factor 1 (IGF-1) deficiency, peripheral resistance to these hormones due to receptor downregulation, impairment of GH/IGF-1 signaling pathways, dysfunction of IGF-binding proteins, reduced IGF-1 bioavailability, and altered expression of genes involved in the GH/IGF axis [12,13,14].

In this context, FMF could be considered as an interesting disease model in which intermittent and recurrent inflammation triggers potentially reversible cytokine-induced GH resistance, providing insights into the dynamic interplay between inflammation and endocrine function. The overall objective of this scoping review is to assess growth-related data in young patients with FMF and summarize the current evidence on growth outcomes in FMF, paying particular attention to the overall impact of inflammation and anti-inflammatory therapies for such patients.

## 2. Materials and Methods

### 2.1. Data Sources

**Figure 1:**
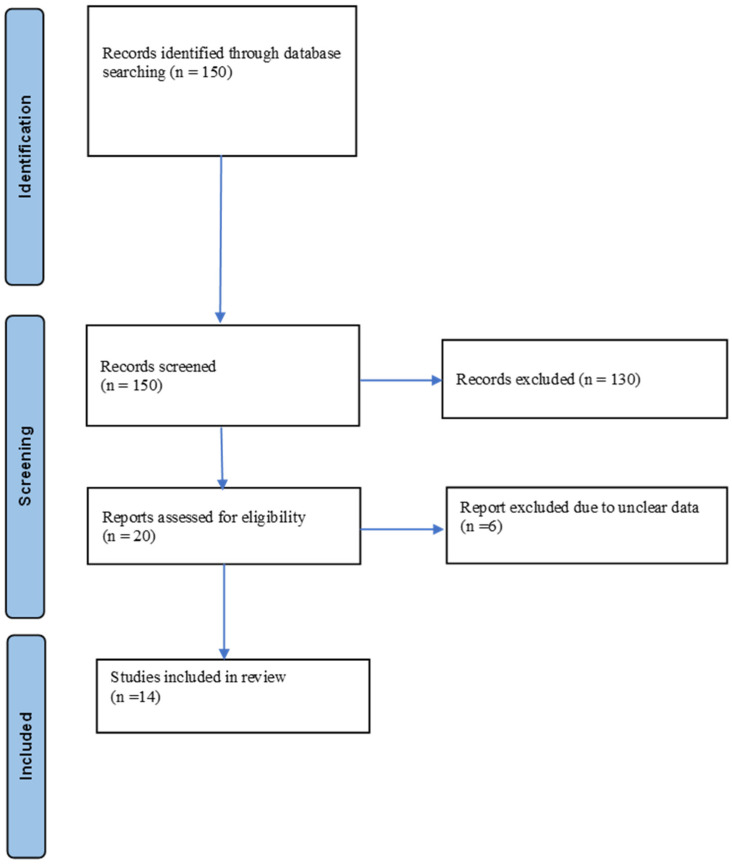
PRISMA 2020 flow diagram revealing the inclusion of medical studies related to children with Familial Mediterranean Fever and growth outcomes within the present review.

We conducted a scoping review without time restrictions, including all studies published up to January 2026, to assess the available evidence on growth outcomes in children with FMF. The search was performed in the PubMed database using the following keywords: “Familial Mediterranean Fever & growth”, “Familial Mediterranean Fever & growth hormone”, “Familial Mediterranean Fever & height”, “Familial Mediterranean Fever & stature”, and “Familial Mediterranean Fever & physical growth”. This scoping review was carried out in accordance with PRISMA (Preferred Reporting Items for Systematic reviews and Meta-Analyses) guidelines to ensure transparent and complete reporting. The recruited studies were eligible if they evaluated: (1) pediatric patients diagnosed with FMF, and (2) growth parameters. We excluded non-English publications and studies that did not assess any growth outcomes. Two independent reviewers (IC and CC) screened titles and abstracts based on these predefined inclusion and exclusion criteria. Full texts of eligible studies were then reviewed, and any disagreements were resolved through open discussion and consensus (with DR). We included both randomized and non-randomized controlled trials as well as observational studies (prospective and retrospective), including cohort, case–control, and cross-sectional studies, along with case series and case reports. The search strategy has been illustrated in Figure 1.

### 2.2. Study Selection

A total of 150 records were initially identified through database searching. During the preliminary screening phase, 40 non-English publications were excluded, along with 20 records for which the full text was not accessible, as well as 40 duplicate entries. Subsequently, 30 articles were eliminated after title and abstract screening because they did not fulfill the predefined inclusion criteria or the objective of this review. Out of the 20 studies that underwent full-text assessment, 6 were further excluded following in-depth evaluation and discussion concerning methodological quality and data reliability. Ultimately, 14 studies finally met all eligibility criteria and were included in the present review. The detailed selection process is illustrated in a PRISMA flow diagram in Figure 1, while a comprehensive summary of the findings is presented in Table 1.

### 2.3. Data Extraction

Data extraction was performed using a standardized template under the direct supervision of the chief pediatric endocrinologist (CC). For each eligible study, the following variables were collected: study design, sample size and mean age of participants, type of treatment, follow-up period and growth outcomes. In addition, methodological limitations and any potential conflicts of interest disclosed by the authors (IC, CC, and DR) were reviewed and discussed when available. A formal ethical approval was not required for this review.

### 2.4. Data Analysis and Presentation

We adhered to the PRISMA guidelines to ensure methodological transparency, rigor, and completeness in reporting. Given the heterogeneity of study designs, populations studied, interventions, and outcomes among the included articles, a quantitative synthesis was not deemed appropriate. Instead, a comprehensive narrative synthesis was undertaken to systematically summarize, compare, and interpret the findings. This approach allowed us to identify key themes, patterns, and gaps in the existing medical literature, while also highlighting areas of inconsistency and divergence across studies.

## 3. Results

Among the 14 studies included in this review [15,16,17,18,19,20,21,22,23,24,25,26,27,28], most were published after 2010, reflecting a general increasing interest in growth outcomes for children with FMF. Seven studies were retrospective, four were prospective, one was a cross-sectional case–control study, one was comparative, and one had an unspecified design. Collectively, the studies analyzed data from approximately 1144 children (1032), including both FMF patients and healthy controls (*n* = 112). Sample sizes ranged from 11 to 350 participants, with the majority of studies including fewer than 100 subjects. Colchicine was the most commonly evaluated treatment, which was investigated in 12 studies, while 3 studies also assessed biologic agents acting on IL-1 oversecretion (canakinumab and/or anakinra). Follow-up duration periods varied widely across studies, although several of them did not report follow-up length.

Growth parameters were within normal ranges in most cases. Two studies including control groups found no significant differences between FMF patients and healthy children for height, growth velocity, bone age, or growth-related biomarkers [16,20]. Similarly, one cross-sectional study reported normal anthropometric Z-scores in all patients [23]. Longitudinal analyses generally demonstrated improvements in HSDS during colchicine therapy, with reported increases from −1.00 to −0.54, from −0.64 ± 1.20 to −0.26 ± 1.07, and from −0.6 to −0.2 [17,19,21,22]. Data on final height were limited but reassuring, with one large study reporting a preserved final height in 83.3% of females and 91.7% of males with FMF [28]. However, findings were not entirely consistent. One large retrospective study reported a significant decrease in HSDS at the end of the follow-up period, particularly in patients carrying ≥1 M694V mutation and in those with moderate-to-severe disease expression, although overall severity was not associated with growth outcomes in most studies [17,21,24]. Regarding additional influencing factors, Türkmen et al. found that inflammatory markers were not associated with growth parameters, while FMF attack rate showed weak, nonsignificant negative correlations with HSDS [18]. In contrast, Cakar et al. and Yoldaş et al. reported that growth improvement was more pronounced in patients without recurrent attacks [21,22]. Savgan-Gürol et al. observed a positive correlation between growth velocity and cumulative colchicine dose, whereas Zung et al. showed that earlier initiation of colchicine was associated with better height outcomes, with a negative correlation between HSDS and age at treatment start [17]. Biologic therapies also showed variable but generally positive effects on growth, with increases in HSDS accompanied by improvement of disease activity and laboratory markers [25,26,27]. Overall, despite some heterogeneity, the current evidence suggests that growth is generally preserved and regular in the vast majority of children with FMF, particularly if on colchicine treatment, and that early therapy initiation and effective overall disease control are crucial for optimal growth outcomes.

## 4. Discussion

Children with chronic illnesses may display poor growth with a short stature in adulthood, depending on the multiple organs involved or effects of specific therapies in combination with nutritional, metabolic, and genetic factors: an impairment of growth should contribute to poor body image, reduced educational attainment, lower adult economic productivity, and increased risk of chronic diseases like diabetes and obesity [29]. Different authors have now documented an impaired GH-IGF-1 axis with an increase in plasma factors that inhibit the effects of IGF-1 in children with chronic illnesses, and this present review aimed to synthesize the available evidence regarding growth outcomes in children with FMF, a genetic defect of innate immunity with many systemic consequences [1,8], with particular attention to the possible interplay between inflammation and linear growth impairment. The association between chronic inflammation and impaired growth has been extensively investigated in other pediatric chronic inflammatory disorders, particularly inflammatory bowel disease and juvenile idiopathic arthritis [30,31,32]. In these conditions, multiple factors may contribute to growth failure, including disease activity, malnutrition, reduced physical activity, exposure to corticosteroids, and persistent systemic inflammation. Elevated pro-inflammatory cytokines such as TNF-α, IL-1, and IL-6 have been implicated in the development of GH resistance, resulting in reduced IGF-1 and IGFBP-3 levels and altered growth plate function [12].

Similarly, FMF is characterized by recurrent inflammatory attacks associated with increased cytokine production, while a state of subclinical inflammation may persist even during attack-free periods in some patients [33,34]. Several studies demonstrated elevated circulating levels of IL-6, IL-12, IL-17, IL-18, TNF-α, IFN-γ, and soluble IL-2 receptor in FMF patients compared with healthy controls, supporting the presence of an ongoing inflammatory milieu [35]. Moreover, cytokine dysregulation appears to be more pronounced in patients carrying high-risk *MEFV* mutations, particularly those with the M694V variant in homozygosis [35,36]. However, although these findings provide a biologically plausible explanation for impaired growth in FMF, the clinical evidence directly linking inflammatory cytokines to dysfunction of the GH–IGF-1 axis remains limited. Only a minority of studies considered by this review performed detailed endocrine evaluations, and longitudinal assessments of IGF-1, IGFBP-3, or dynamic GH testing were rarely available. Therefore, the concept of FMF as a model of cytokine-induced GH resistance should currently be considered mainly hypothetical and supported predominantly by indirect evidence extrapolated from other chronic inflammatory diseases. In contrast, stronger clinical associations emerged for factors such as disease severity, persistent inflammatory activity, delayed treatment initiation, colchicine responsiveness, treatment adherence, and high-risk *MEFV* genotypes. In particular, patients with moderate-to-severe disease phenotypes or M694V mutations appeared more likely to experience less favorable growth outcomes, suggesting that chronic inflammatory burden rather than isolated endocrine dysfunction may represent the main determinant of impaired growth in pediatric FMF. In particular, patients carrying at least one M694V mutation or presenting with moderate-to-severe disease activity showed lower baseline and follow-up height HSDS, supporting the hypothesis that a higher inflammatory burden may subtly impair growth velocity and final height attainment [23]. The relationship between genotype and growth outcomes, however, remains somewhat controversial. While Türkmen et al. reported that homozygous M694V mutations did not significantly affect weight or height SDS, more recent experiences support a possible prognostic role of specific *MEFV* mutations in growth impairment [18]. Kişla Ekinci et al. demonstrated that children carrying at least one M694V mutation had significantly lower initial HSDS values compared with patients without this mutation. Interestingly, despite poorer baseline auxological parameters, these patients showed substantial catch-up growth following appropriate and sustained colchicine treatment, suggesting that early control of inflammation may partially reverse the negative impact of severe genotypes on growth outcome [24]. Additional evidence supports the association between M694V mutations and a more severe inflammatory phenotype in pediatric FMF. In fact, patients carrying homozygous or compound heterozygous M694V mutations showed higher disease severity scores and persistently elevated inflammatory markers, suggesting that peculiar genotypes may contribute to a greater chronic inflammatory burden, which could potentially influence long-term growth outcomes despite apparently preserved growth parameters in some cohorts [37].

Overall, these findings underline the importance of considering both genetic background and disease severity when evaluating growth patterns in pediatric FMF patients, as they may contribute not only to disease prognosis but also to long-term developmental outcomes. Across 14 studies included by this review, in general terms, growth can be judged regular for children with FMF, especially if fully compliant with colchicine therapy, although some variability exists depending on genetics, disease severity, and treatment starting time. Growth parameters, such as HSDS, showed largely normal values, considering FMF patients and comparing them to healthy peers. Moreover, consistent improvements of HSDS were documented in children under treatment, including increases from −1.00 to −0.54, −0.64 ± 1.20 to −0.26 ± 1.07, and −0.6 to −0.2 [16,18,20,21]. Final height data, although limited, were reassuring: one large study reported preserved final height in 83.3% of females and in 91.7% of males, suggesting that linear growth is largely maintained if systemic inflammation is effectively under control [27]. Colchicine is essential for ensuring growth preservation, with the aim of modulating cytokine secretion and pyrin inflammasome activation and decreasing the negative impact of recurrent inflammation on growth plates [2,3,5,7,9]. Several studies demonstrated that early initiation of colchicine and higher cumulative doses of colchicine were positively correlated with growth velocity, while delays in starting colchicine treatment were associated with lower outcomes [15,16].

Long-term blockade of IL-1 has been found to restore the clinical equilibrium in many systemic autoinflammatory syndromes of childhood, and IL-1 inhibitors have become cardinal weapons in managing both monogenic innate immunity defects and a plethora of polygenic diseases occurring in children, including Still’s disease and Kawasaki disease [38,39,40]. Biologic therapies targeting IL-1β, including canakinumab and anakinra, have been studied in smaller studies and were generally associated with increases in HSDS and height percentiles, along with improvements in laboratory markers and disease activity [24,25,26]. These findings underscore the central role of IL-1β in mediating growth impairment and support the concept that a specific targeted cytokine blockade may be beneficial in colchicine-resistant patients or in the most severe cases. Interestingly, some patients showed no significant height change after biologic therapy, suggesting that additional factors, such as duration of prior inflammation and individual endocrine responsiveness, may also influence growth outcomes [26].

**Figure 2:**
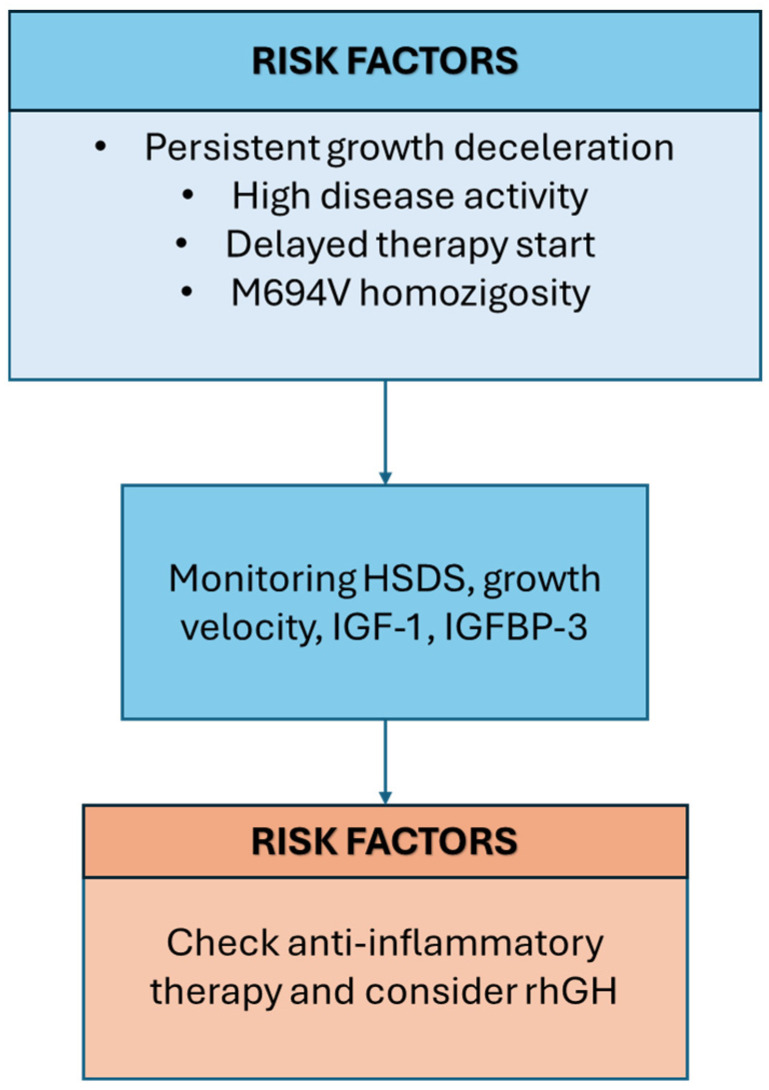
Algorithm for endocrine workup and evaluation of risk factors for stunted growth in children with Familial Mediterranean Fever, based on expert opinion and available evidence. Insulin-like Growth Factor 1 (IGF-1), Insulin-like Growth Factor Binding Protein 3 (IGFBP-3), Height Standard Deviation Score (HSDS), and Recombinant Human Growth Hormone (rhGH).

A proposed expert-based endocrine workup algorithm for the evaluation of risk factors associated with impaired growth in pediatric patients with FMF, developed on the basis of the available evidence identified in this scoping review, is presented in Figure 2. The algorithm integrates clinical, genetic, and inflammatory parameters that have been associated with suboptimal auxological outcomes, including disease severity, persistence of subclinical inflammation, treatment adherence, responsiveness to colchicine with adherence to treatment, and the presence of high-risk *MEFV* genotypes. Particular attention is given to patients carrying the M694V variant, in whom a higher inflammatory burden and more severe disease phenotypes have been consistently reported. The proposed diagnostic pathway also emphasizes the role of targeted endocrine assessment in selected cases, including evaluation of the GH–IGF-1 axis when clinically indicated, in order to differentiate inflammation-related growth impairment from a primary endocrine dysfunction. This structured approach aims to support early identification of children at increased risk of growth failure and to guide timely interventions that may optimize growth outcomes in children with FMF.

## 5. Limitations of the Study

Despite encouraging results, several limitations of our review should be noted. Most studies evaluated in this scoping review were small, single-center, or retrospective, with heterogeneous follow-up periods and variable reporting of disease severity, FMF attack frequency, and phenotype-genotype correlations. Data on growth and final adult height remained sparse, and standardized growth assessments were not uniformly applied. In addition, the lack of longitudinal endocrine assessments limits the understanding of the dynamic and potentially reversible nature of GH resistance in FMF. Moreover, a limitation of this review is that the literature search was conducted exclusively using the PubMed database; therefore, potentially relevant studies indexed in other databases may have been missed.

Prospective multi-center studies with longer follow-up and integration of endocrine biomarkers are needed to better define the long-term impact of FMF and disease management on children’s growth and final adult stature.

## 6. Conclusions

Management of children with FMF requires a collaborative multidisciplinary approach involving many healthcare professionals across different clinical settings [41,42,43,44]. The available evidence suggests that growth outcomes in children with FMF are heterogeneous and influenced by multiple factors, including disease severity, inflammatory control, treatment adherence, and genetic background. While many patients seem to maintain normal growth patterns under adequate anti-inflammatory treatment, some studies have reported reduced HSDS over time and less favorable auxological outcomes, particularly among patients with more severe disease phenotypes or high-risk *MEFV* mutations such as M694V. Persistent or recurrent inflammation may contribute to subtle impairment of linear growth despite apparent clinical control, whereas early and effective treatment with colchicine appears to play a key role in preserving normal growth trajectories. These findings highlight the importance of early diagnosis, timely initiation of therapy, strict control of inflammatory activity, and close auxological monitoring, especially for patients considered at higher risk for growth impairment. Nevertheless, the available data regarding final adult height and long-term endocrine outcomes remain limited and partially inconsistent because of the heterogeneity and methodological limitations of the published studies. Further prospective longitudinal studies integrating detailed endocrine and genetic assessments are warranted to better clarify the long-term impact of FMF and its treatment on growth trajectories and adult height attainment.

## Figures and Tables

**Table 1:** List of the medical studies related to growth outcomes in children with Familial Mediterranean Fever. Familial Mediterranean Fever (FMF), Insulin-like Growth Factor 1 (IGF-1), Insulin-like Growth Factor Binding Protein 3 (IGFBP-3), Height Standard Deviation Score (HSDS), NA (not applicable).

Study	Zemer et al. (1991) [15]	Savgan-Gürol et al. (2001) [16]	Zung et al. (2006) [17]	Türkmen et al. (2008) [18]	Ozçakar et al. (2010) [19]	Kosan et al. (2013) [20]	Cakar et al. (2013) [21]	Yoldaş et al. (2016) [22]	Zaki et al. (2018) [23]	Kişla Ekinci et al. (2019) [24]	Balci et al. (2020) [25]	Pinchevski-Kadir et al. (2023) [26]	Aydin et al. (2024) [27]	Bayrak Demirel et al. (2025) [28]
**Design**	Clinical study	Prospective study	Comparative study	Retrospective study	Retrospective study	Prospective study	Prospective study	Prospective study	Cross–sectional case–control study	Retrospective study	Retrospective study	Retrospective study	Retrospective study	Retrospective study
**Sample size (N)**	350	93 (51 patients with FMF, 42 healthy children)	30	33	50	65 (30 patients with FMF, 35 healthy children)	51	51	85 (50 patients with FMF, 35 healthy children)	126	11	22	37	140
**Mean age (years)**	NA	NA	4.32 ± 1.82	7.1 ± 2.6	6.5	8.19 ± 1.97	6.4	6.4 ± 2.39	7.0 ± 2.0	7.3 ± 3.6	NA	12.85 ± 4.2	3.6–10.1	8–18
**Treatment**	Colchicine	Colchicine	Colchicine	Colchicine	Colchicine	Colchicine	Colchicine	Colchicine	Colchicine	Colchicine	Colchicine and canakinumab	Anakinra and/or canakinumab	Canakinumab	Colchicine
**Follow-up**	NA	NA	2.58 ± 1.55 years	46.2 +/− 39.8 months	3.6 (1–12.5) years	1 year	1 year	1 year	NA	74.7 months (from 7.5 to 169 months)	1.59 (0.56–4.33) years	3.05 ± 1.75 years	NA	NA
**Results**	No growth abnormalities were observed in treated patients; at age 17, those on long-term colchicine were taller than the untreated ones	No differences in height, growth velocity, bone age, target height, or IGF-1 levels; growth velocity was positively correlated with the cumulative colchicine dose	HSDS improved from −1.00 to −0.54, with growth unaffected by disease severity, but influenced by the timing of treatment initiation	Attack rate showed a weak, non-significant negative correlation with HSDS, which remained stable during therapy (from 0.20 ± 1.10 to 0.19 ± 1.19)	Mean HSDS increased from −0.19 +/− 1.01 to 0.13 +/− 0.99.	All patients had normal height percentiles at baseline, with no significant differences from controls in growth measures, anthropometric parameters, or IGF-1 and IGFBP-3 levels at baseline or during follow-up	HSDS increased from −0.6 to −0.2, with greater increases in patients without attacks; disease severity had no effect on growth	HSDS increased from −0.64 ± 1.20 to −0.26 ± 1.07; growth improvement was significant only in patients without attacks	The mean Z-scores of anthropometric measurements were within normal ranges	Mean HSDS was lower at the last visit compared to pre-colchicine values; patients with ≥1 M694V mutation and those with moderate-to-severe disease had lower height at both baseline and follow-up than others.	After canakinumab treatment, HSDS increased significantly from −0.35 to 0.04 SDS, along with improvements in laboratory markers and disease activity	Height percentile increased from 19.6 ± 16% to 30.8 ± 23% after treatment	After treatment, height remained unchanged	Final height is largely preserved in children with FMF (83.3% females and 91.7% males)

## Data Availability

No new data were created or analyzed in this study.

## Abbreviations

The following abbreviations are used in this manuscript:

FMF	Familial Mediterranean fever
IL	Interleukin
TNF	Tumor necrosis factor
IFN	Interferon
GH	Growth hormone
IGF	Insulin-like growth factor
IGFBP	Insulin-like growth factor binding protein
HSDS	Height standard deviation scores
PRISMA	Preferred reporting items for systematic reviews and meta-analyses
